# Acquisition-dependent modulation of hippocampal neural cell adhesion molecules by associative motor learning

**DOI:** 10.3389/fnana.2022.1082701

**Published:** 2022-12-21

**Authors:** Juan D. Navarro-López, Ana Contreras, Katia Touyarot, Ana I. Herrero, César Venero, Karine Cambon, Agnés Gruart, José M. Delgado-García, Carmen Sandi, Lydia Jiménez-Díaz

**Affiliations:** ^1^Laboratory of Neurophysiology and Behavior, Facultad de Medicina de Ciudad Real, Universidad de Castilla-La Mancha, Ciudad Real, Spain; ^2^INRAE, Bordeaux INP, NutriNeuro, University of Bordeaux, Bordeaux, France; ^3^Department of Psychobiology, Universidad Nacional de Educación a Distancia, Madrid, Spain; ^4^Direction de la Recherche Fondamentale (DRF), Institut François Jacob, MIRCen, Commissariat à l’Energie Atomique et aux Energies Alternatives (CEA), Fontenay-aux-Roses, France; ^5^Division of Neurosciences, Pablo de Olavide University, Seville, Spain; ^6^Laboratory of Behavioral Genetics, Brain Mind Institute, School of Life Sciences, École Polytechnique Fédérale de Lausanne (EPFL), Lausanne, Switzerland

**Keywords:** classical eyeblink conditioning, synaptosomes, L1, NCAM, PSA-NCAM, hippocampus, associative motor learning

## Abstract

It is widely accepted that some types of learning involve structural and functional changes of hippocampal synapses. Cell adhesion molecules neural cell adhesion molecule (NCAM), its polysialylated form polysialic acid to NCAM (PSA-NCAM), and L1 are prominent modulators of those changes. On the other hand, trace eyeblink conditioning, an associative motor learning task, requires the active participation of hippocampal circuits. However, the involvement of NCAM, PSA-NCAM, and L1 in this type of learning is not fully known. Here, we aimed to investigate the possible time sequence modifications of such neural cell adhesion molecules in the hippocampus during the acquisition of a trace eyeblink conditioning. To do so, the hippocampal expression of NCAM, PSA-NCAM, and L1 was assessed at three different time points during conditioning: after one (initial acquisition), three (partial acquisition), and six (complete acquisition) sessions of the conditioning paradigm. The conditioned stimulus (CS) was a weak electrical pulse separated by a 250-ms time interval from the unconditioned stimuli (US, a strong electrical pulse). An acquisition-dependent regulation of these adhesion molecules was found in the hippocampus. During the initial acquisition of the conditioning eyeblink paradigm (12 h after 1 and 3 days of training), synaptic expression of L1 and PSA-NCAM was transiently increased in the contralateral hippocampus to the paired CS-US presentations, whereas, when the associative learning was completed, such increase disappeared, but a marked and bilateral upregulation of NCAM was found. In conclusion, our findings show a specific temporal pattern of hippocampal CAMs expression during the acquisition process, highlighting the relevance of NCAM, PSA-NCAM, and L1 as learning-modulated molecules critically involved in remodeling processes underlying associative motor-memories formation.

## 1 Introduction

Acquired motor abilities are believed to be stored in the form of structural and/or functional changes in synaptic efficiency (Hebb, 1949; Kandel, 2001). The neural cell adhesion molecule (NCAM) and the L1, two members of the immunoglobulin superfamily of cell adhesion molecules (CAM), are important constituents of synapses that mediate neuron-neuron adhesion and shape the formation of neuronal networks during brain development and synaptic plasticity throughout adulthood (Duncan et al., 2021). Both molecules also play a key role in regulating the structural synaptic changes underlying learning acquisition and memory formation (Sandi, 2004; Bisaz et al., 2009; Conboy et al., 2010; Hartz and Rønn, 2010; Duncan et al., 2021). In addition, post-translational attachment of polysialic acid to NCAM (PSA-NCAM), a reaction shown to downregulate NCAM adhesion properties (Rutishauser and Landmesser, 1996; Colley, 2010; Hildebrandt et al., 2010), has also been implicated in synaptic remodeling after learning experiences (O’Malley et al., 2000; Kiss et al., 2001; El Maarouf and Rutishauser, 2010; Gascon et al., 2010; Saini et al., 2020).

The role of NCAM, its polysialylated form, and L1 in synaptic plasticity and cognitive processes has been based on several lines of evidence. First, interference with the expression and/or function of these molecules in knock-out mice models or pharmacologically by the administration of antibodies, peptides, enzymes, or antisense oligonucleotides results in impaired long term potentiation (LTP) (Cremer et al., 2000; Eckhardt et al., 2000; Duncan et al., 2021) and learning and memory deficits (Cremer et al., 1994; Arami et al., 1996; Becker et al., 1996; Cambon et al., 2003; Seymour et al., 2008; Bisaz et al., 2009). Second, the converse evidence, i.e., improved LTP and enhanced learning abilities, has been achieved by mimicking NCAM function (Cambon et al., 2004) or by L1 or PSA-NCAM over-expression (Wolfer et al., 1998; Rendeiro et al., 2014; Saini et al., 2020). Third, mRNA and protein expression of NCAM, PSA-NCAM, and L1 in different brain areas is regulated by learning and LTP (Ronn et al., 2000; Hartz and Rønn, 2010; Saini et al., 2020; Duncan et al., 2021). Specifically, the expression of these CAMs is modulated in the hippocampus 1–24 h after LTP induction, by training in a memory task (Fazeli et al., 1994; Schuster et al., 1998; Ronn et al., 2000), or after a variety of hippocampal-dependent training experiences including passive avoidance training (Doyle et al., 1992a; Fox et al., 1995, 2000; Brandewiede et al., 2014), contextual fear conditioning (Merino et al., 2000; Sandi et al., 2003; Maćkowiak et al., 2009), and olfactory (Knafo et al., 2005a,b), and spatial (Sandi, 2004; Venero et al., 2006; Yamagishi et al., 2018) learning tests. Furthermore, pathologies characterized by learning and memory deficits, such as Alzheimer’s disease, major depression, or drug addiction, are related to a decreased expression of these CAMs (Maćkowiak et al., 2009; Gilabert-Juan et al., 2012; Murray et al., 2018).

Classical conditioning of eyelid responses is a well-known and widely used associative motor learning task. In trace eyeblink conditioning paradigms, the conditioned stimulus (CS) is separated from the unconditioned stimulus (US) presentation by a stimulus-free interval (trace), and the hippocampus is thought to play a major role in the formation of this memory trace (Solomon et al., 1986; Gruart et al., 2006, 2015). In fact, acquisition and retention of trace conditioning requires an intact hippocampus (Weiss et al., 1999; Gruart et al., 2015) and it has been demonstrated that hippocampal synaptic strength is modulated by this type of associative learning (Gruart et al., 2006, 2015).

Given the critical role of the hippocampus in associative learning (Gruart et al., 2015), and the established involvement of NCAM, PSA-NCAM, and L1 in some learning processes (Duncan et al., 2021), we aimed to investigate the time sequence of such neural cell-adhesion molecules modifications during the acquisition of a trace eyeblink conditioning in alert behaving rats.

## 2 Materials and methods

### 2.1 Animals

Fifty-seven adult male Wistar rats (250–300 g before surgery; Iffa-Credo, France), group housed three per cage, were used for all experiments. Animals were kept in their home cages at 22 ± 2°C and with a 12 h light-dark cycle (lights on at 7:00 a.m.). Food and water were provided *ad libitum*. All experimental procedures were reviewed and approved by the Ethical Committee for Use of Laboratory Animals of the National Distance Education University (UNED) and conducted according to the European Union guidelines (2010/63/EU) and the Spanish regulations for the use of laboratory animals in chronic experiments (RD 53/2013 on the care of experimental animals: BOE 08/02/2013). All efforts were made to minimize animal suffering.

### 2.2 Surgery

**FIGURE 1:**
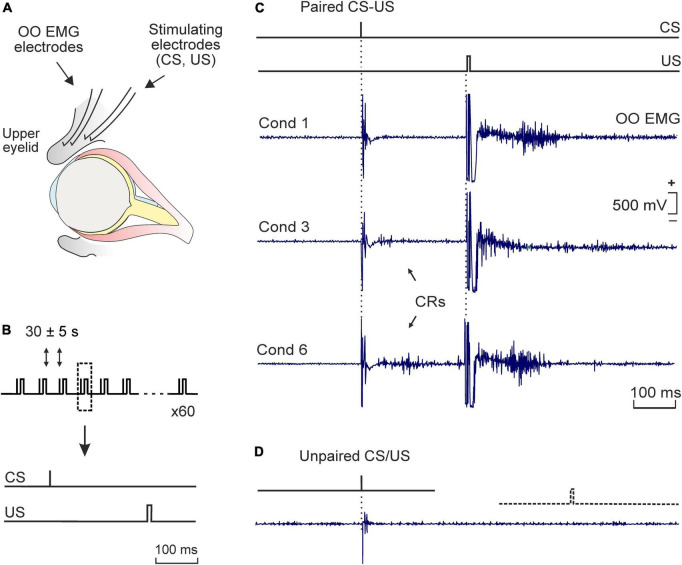
Experimental design. **(A)** Bipolar hook electrodes were implanted on the right supraorbital nerve for electrical stimulation of this sensory nerve. The electromyographic activity of the ipsilateral orbicularis oculi muscle [orbicularis oculi (OO) electromyographic (EMG)] was also recorded. **(B)** Each conditioning session consisted of 6 blocks comprising 10 trails per block. The conditioned (CS) and unconditioned stimuli (US) consisted of square, cathodic pulses presented to the supraorbital nerve, lasting 0.05 ms (1.5X threshold) and 0.5 ms (3X threshold), respectively. The CS was followed by the US with an interval of 250 ms. The CS was presented alone the first trial of each block. **(C)** Representative examples of OO EMG profiles of eyelid responses (in mV) recorded during the 1st, 3rd, and 6th conditioning sessions (cond 1, 3, and 6). Conditioned responses (CRs) are indicated by arrows. **(D)** A typical eyelid response collected during pseudo-conditioning (i.e., unpaired presentation of the CS and the US). Calibration in panel **(C)** also applies to panel **(D)**.

All surgical procedures were carried out under sodium pentobarbital anesthesia (40 mg/kg i.p.) and using aseptic surgical techniques. Four teflon-coated stainless-steel wires (No. 7910; A-M Systems, US) were implanted in the subcutaneous tissue of the right upper eyelid to be used as bipolar stimulating electrodes of the right supraorbital branch of the trigeminal nerve and as bipolar electromyographic (EMG) recording electrodes in the ipsilateral orbicularis oculi (OO) muscle (Figure 1A). Electrode tips were cleaned of the coating cover for ∼0.5 mm and bent as a hook to facilitate stable insertion in the eyelid tissue. Electrodes were soldered to a four-pin connector that was secured to the skull with stainless-steel screws and a dental acrylic resin. Details of this surgical preparation have been described elsewhere (Jiménez-Díaz et al., 2006; Valenzuela-Harrington et al., 2007). A recovery period of 7–9 days was allowed before any behavioral experiment.

### 2.3 Classical eyeblink conditioning training

Classical conditioning training was developed using an experimental design that allowed the animals to freely move during acquisition sessions (i.e., movement restrictions were avoided and animals freely moved during CS-US presentations in each training session). For this purpose, electrical stimulation of the supraorbital sensory nerve was used as conditioned (CS) and unconditioned (US) stimuli, respectively (Figure 1). Electrical stimulation elicited reflex eyelid movements associated with the presence of reflex responses in the OO EMG that were recorded for learning quantification purposes [i.e., for conditioned responses (CRs) measurement]. For recordings, each rat was placed in a plastic cage (30 × 20 × 20 cm). Prior to behavioral training, animals were handled daily for 2 min during 3 consecutive days and habituated for 2 more min to the specific recording procedure. A tether, containing a low-weight, 4-channel wire (to relay OO EMG activity and to deliver the CS and US stimuli), was connected to the socket implanted on the animal’s head and attached to a 360° rotatory electrical commutator (electrical *swivel*, Plastics One, US) which allowed the rat to move freely during training.

Classical conditioning was achieved using a trace paradigm (Figure 1B). Briefly, a short (50 μs), weak (∼1.5 × threshold, defined as the minimum intensity able to elicit a small eyelid movement associated with the presence of a reflex response in the OO EMG), square, cathodal pulse was presented as CS. The US consisted of a long (500 μs), strong (∼2–3 × threshold, able to consistently elicit a complete and immediate eyelid closure), square, cathodal pulse. The US started 250 ms after the end of the CS. CS and US intensities (in mA) were experimentally determined for each subject before the beginning of the training and remained unchanged along the conditioning program. In total, two habituation and six conditioning sessions were carried out per animal. During habituation, the CS was presented alone. A conditioning session consisted of 60 paired CS-US presentations, and lasted ∼30 min. In 10% of the cases, the CS was presented alone. CS-US presentations were separated at random by 30 ± 5 s. For control purposes, pseudo-conditioning and context-exposed groups were also included in the experimental design. For pseudo-conditioning, unpaired CS and US presentations were carried out for 6 sessions (60 CS and 60 US per session, presented at random). For context-exposure, rats were placed into the recording chamber for 30 min, in the absence of any stimulation. Further details of this experimental preparation are presented elsewhere (Fontan-Lozano et al., 2005; Inda et al., 2005).

### 2.4 Electromyography recording

The EMG activity of the OO muscle was recorded using a GRASS P511 differential amplifier with a bandwidth of 1 Hz to 10 kHz (Grass-Telefactor, USA). Data was stored directly on a computer with an analog/digital converter (1401 Plus; Cambridge Electronic Design, UK), at a sampling frequency of 11–22 kHz and an amplitude resolution of 12 bits. Data was analyzed off-line for quantification CRs. The presence of EMG activity during the CS-US period which lasted >10 ms and was initiated >50 ms after CS onset was considered a CR; thus, we avoided including putative alpha responses in the quantification of true CRs. Those recordings presenting EMG activity in the 250 ms preceding CS presentation (change in baseline), or with an evident startle response (response beginning 4 ms after CS and expanded until 100 ms) or artifactual recordings were excluded from the quantitative analysis. The percentage of trials containing a CR in a given session was calculated based on the number of valid trials.

### 2.5 Tissue preparation

Animals were anesthetized with an overdose of 2,2,2-tribromoethanol (Sigma-Aldrich, US) 12 h after the end of the 1st, 3rd, or 6th training session and decapitated. A total of 4–9 animals per group were used. Immediately after decapitation, brains were kept on ice and both right and left hippocampi (ipsi- and contralateral to stimuli presentation, respectively) were dissected. Tissue samples were coded and stored at −80°C until use. Crude synaptosomal pellets were obtained according to a modified protocol from Lynch and Voss (1991). In brief, tissue was homogenized in ten volumes of ice-cold sucrose (0.32 M) and HEPES (5 mm) buffer that contained a cocktail of protease inhibitors (Complete TM; Boehringer Mannheim, UK) with 16 strokes, and centrifuged at 1,000 *g* for 5 min. The supernatant was then centrifuged at 15,000 *g* for 15 min, and the pellet was resuspended in Krebs buffer, containing protease inhibitors, for further use. Protein concentration for each sample was measured by the Bradford method (Bradford, 1976).

### 2.6 ELISAs

Total NCAM (including all NCAM isoforms), PSA-NCAM, and L1 were quantified by enzyme-linked immunosorbent assays (ELISAs) according to a previously described protocol (Merino et al., 2000). In brief, flat bottom 96 well microplates were allowed to adsorb a coating solution (Na_2_CO_3_ 0.1 M/NaHCO_3_, 0.1 M) for 2 h at room temperature (RT). The solution was removed and 50 μl of pellet samples were added at a concentration of 10 μg/ml to each well of polystyrene flat-bottom ELISA plates. Plates were incubated overnight at 4°C and then washed three times with 1 M phosphate-buffered saline (PBS) containing 0.05% Tween 20, pH 7.4. Additional binding sites were blocked with BSA (3%) for 2 h at RT. Wells were rinsed three times as described above, and incubated with 50 μl aliquots of the corresponding first antibody for 20–24 h at 4°C. Then, wells were washed and 50 μl aliquots of peroxidase-conjugated secondary antibody were added for a 2 h incubation period. Afterward, 50 μl of citrate buffer (50 mm Na_2_HPO_4_, 25 mm citric acid, pH 4.5) containing 1 mg/ml o-phenylene diamine and 0.06% H_2_O_2_, added just before use, were placed in each well, and the peroxidase allowed to react for 10 min at RT. The reaction was terminated by the addition of 50 μl of 10 M H_2_SO_4_ to each well. Optical density was determined by measuring absorbance at 492 nm with a Microplate Reader (DigiScan Reader V3.0 and DigiWIN software; ASYS Hitech GmbH, Austria).

The following primary antibodies were used: a polyclonal rabbit anti-rat NCAM IgG (1:300; from Prof. Elisabeth Bock, University of Copenhagen, Denmark); a monoclonal mouse IgM antibody that recognizes specifically a-2–8-linked PSA with chain length superior to 12 residues, and binds with high specificity to PSA on NCAM (1:2 dilution of ascites fluid; diluted 1:300 for the ELISA; Men B, clone B1.2; generous gift from Prof. Genevieve Rougon, CNRS Marseille, France); and a monoclonal rat anti-rat L1 IgG (1:150; Chemicon International, US). As secondary antibodies, an anti-rabbit IgG peroxidase conjugate (whole molecule conjugate; 1:2000; Jackson Immunoresearch, US), an IgM anti-mouse peroxidase conjugate (l chain; 1:1000; Sigma-Aldrich, US), and an anti-rat Ig-POD Fab fragments (from sheep immunoglobulin) to IgG-rat peroxidase conjugated (1:500; Boehringer, UK) were used, respectively.

### 2.7 Statistical analysis

Data was expressed as mean ± SEM. Statistical differences between percentages of CRs were determined by repeated measures two-way ANOVA, followed by Tukey’s *post-hoc* analysis. Biochemical data were analyzed with three-way ANOVAs (using training, sessions, and hippocampal side as fixed factors), followed by Tukey’s *post-hoc* analysis. Statistical significance was set at *p* < 0.05. If the Levene’s test for normal distribution was significant then data were normalized by square root transformation (percentage of conditioned responses). All analyses were performed using the SPSS software v.28 (RRID:SCR_002865; IBM, US) and final figures were prepared using CorelDraw X8 Software (RRID:SCR_014235; Corel Corporation, Canada).

## 3 Results

### 3.1 Classical eyeblink conditioning acquisition

Animals were randomly assigned to one of the following three experimental paradigms: classical eyeblink conditioning (paired group, *n* = 26), pseudo-conditioning (unpaired group, *n* = 18), or context exposure without stimulation (context group, *n* = 13). Animals were sacrificed after the 1st (paired group *n* = 8; unpaired group *n* = 6; context group *n* = 4), 3rd (paired group *n* = 9; unpaired group *n* = 6; context group *n* = 5), or 6th (paired group *n* = 9; unpaired group *n* = 6; context group *n* = 4) training sessions.

For conditioned and pseudo-conditioned animals, we first confirmed that electrode implantation in the upper eyelid did not disturb reflexively evoked eyeblink responses. We found that the electrical stimulation (2X threshold) of the supraorbital nerve evoked an early (6–7 ms) activation of the OO muscle, followed by a second, more variable (15–25 ms) EMG activation (not illustrated). These successive muscle activations corresponded to the R1 and R2 components previously described in rats (Valenzuela-Harrington et al., 2007), as well as humans (Kugelberg, 1952), cats (Gruart et al., 1995), and mice (Dominguez-Del-Toro et al., 2004).

**FIGURE 2:**
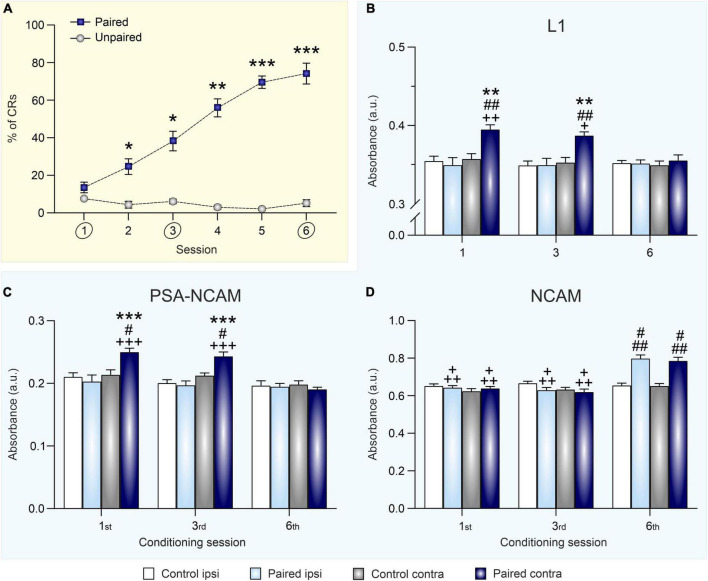
Eyeblink conditioning acquisition modulates synaptic expression of L1, PSA-NCAM, and NCAM in the hippocampus. **(A)** Percentage of conditioned eyelid responses [conditioned responses (CRs)] across six conditioning sessions. Data is expressed as mean ± SEM of animals sacrificed at the end of the sixth session. *p < 0.05, **p < 0.01, ***p < 0.001 vs. unpaired. **(B–D)** Optical density measurements of L1 **(B)**, PSA-NCAM **(C)**, and NCAM **(D)** expression, after the 1st, 3rd, and 6th training sessions, in hippocampal synaptosomes obtained from right (ipsilateral to CS and US presentation) and left (contralateral) hippocampi of conditioned and control (merged from unpaired and context groups) animals. Data is expressed as mean ± SEM for each session (1st: paired group *n* = 8 and control group *n* = 10; 3rd: paired group *n* = 9 and control group *n* = 11; 6th: paired group *n* = 9, and control group *n* = 10). a.u., arbitrary units. **p < 0.01, ***p < 0.001 *vs.* ipsi; ^#^*p* < 0.05, ^##^*p* < 0.01 vs. control; ^+^*p* < 0.05, ^+ +^*p* < 0.01, ^+ + +^*p* < 0.001 vs. session 6.

Then the learning capabilities of conditioned and pseudo-conditioned rats were compared using a trace conditioning paradigm. As illustrated in Figure 1C, conditioned eyelid responses reaching criterion were evident by the 3rd conditioning session, although they were already frequently observed during the second session (Figure 2A). Data showed that conditioned animals presented the normal learning curve previously described in rodents when using a similar trace conditioning procedure (Gruart et al., 2006; Valenzuela-Harrington et al., 2007; Fernandez-Lamo et al., 2009). Animals sacrificed after one conditioning session (initial acquisition) presented a mean of 14 ± 3% CRs, while those sacrificed after the third session (partial acquisition) had already reached a mean of 38 ± 5% CRs. Animals trained for six sessions (complete acquisition) had reached asymptotic values of 74 ± 6% CRs (Figure 2A). By contrast, pseudo-conditioned animals (i.e., those receiving the unpaired presentation of the stimuli used as CS and US; Figure 1D) presented extremely poor learning performances, showing a low rate (<8%) of eyelid responses that fulfilled the criterion set for OO EMG to be considered as CRs (Figures 1D, 2A). When comparing the percentage of CRs obtained from conditioned versus pseudo-conditioned control animals, a two-way ANOVA showed a significant training difference [*F*_(1,8)_ = 318.802; *p* < 0.001; Figure 2A], and *post-hoc* analysis revealed that the different was present in all training sessions except the first one.

### 3.2 Hippocampal synaptic NCAM, PSA-NCAM, and L1 expression during classical eyeblink conditioning is acquisition-dependent

In order to evaluate the effect of classical eyeblink conditioning on the expression of neural cell adhesion molecules in the hippocampus, expression levels of NCAM, PSA-NCAM, and L1 in hippocampal crude synaptosomes were determined 12 h after the 1st (initial acquisition), 3rd (partial acquisition), and 6th conditioning sessions (complete acquisition) (Figures 2B–D).

The two control groups submitted to either pseudo-conditioning (unpaired CS/US training) or context exposure (non CS or US stimulation) did not significantly differ in neither their synaptic ipsi- nor the contralateral hippocampal NCAM [*F*_(1,42)_ = 1.967; *p* = 0.168], PSA-NCAM [*F*_(1,50)_ = 0.553; *p* = 0.461], or L1 [*F*_(1,48)_ = 0.066; *p* = 0.79] expression levels at any of the training session studied (Supplementary Table 1). Thus, for presentation and statistical purposes, Figures 2B–D show the merge data from pseudo-conditioned and context groups, as data obtained from both control groups were equivalent at all testing times.

Regarding L1 levels (Figure 2B), a three-way ANOVA showed a significant training effect [*F*_(1,98)_ = 11.00; *p* = 0.0013] and *post-hoc* analysis indicated that it was specifically an upregulation of L1 expression in the contralateral hippocampus of conditioned rats after sessions one and three. Moreover, there was also an hippocampal side effect [*F*_(1,98)_ = 16.92; *p* < 0.0001], since that increase in the contralateral hippocampus was not present in the ipsilateral area, and a session effect [*F*_(2,98)_ = 3.671; *p* = 0.029] as the difference between groups was lost in the last session. Thus, L1 levels seems to be regulated by conditioning learning in an acquisition-dependent way in the contralateral hippocampus, being overexpressed specifically in this side during initial and partial learning acquisition but not when the conditioning was completely acquired.

A similar pattern of learning-induced modulation was found for synaptic PSA-NCAM levels in the paired (i.e., conditioned) group (Figure 2C). Conditioning significantly upregulated PSA-NCAM expression [training effect: *F*_(1,102)_ = 4.113; *p* = 0.0452] in the contralateral hippocampus after one and three training sessions. Once again, there was a significant side effect [*F*_(1,102)_ = 21.51; *p* < 0.0001], specifically in the contralateral hippocampus. After one and three conditioning session PSA-NCAM values were enhanced, but they did not differ to control ones after six sessions [session effect: *F*_(2,102)_ = 14.59; *p* < 0.0001]. As it happened with L1, PSA-NCAM was overexpressed in the contralateral hippocampus during the initial and partial acquisition sessions but decreased to normal values after complete conditioning acquisition.

Finally, total NCAM expression in hippocampal crude synaptosomes, as assessed by ELISA using an antibody that recognizes all NCAM isoforms, was bilaterally increased 12 h after submitting rats to six conditioning sessions [Figure 2D; session effect: *F*_(2,91)_ = 47.53; *p* < 0.0001]. The regulation was not only session-dependent, but also training-dependent. Hence, synaptic NCAM levels from conditioned rats were higher than those of both pseudo-conditioned and context-exposed control groups after the sixth conditioning session, when asymptotic learning levels had been reached [training effect: *F*_(1,91)_ = 21.23; *p* < 0.0001]. As shown in Figure 2D, NCAM levels from conditioned rats were equivalent between both hippocampi across conditioning [side effect: *F*_(1,91)_ = 3.195; *p* = 0.0772]. Therefore, NCAM seems to be bilaterally upregulated by conditioning learning only after complete acquisition of the association.

## 4 Discussion

### 4.1 Cell adhesion molecules upregulation is specific to conditioned rats

The hippocampus has been one of the brain areas tightly related to the acquisition of new motor abilities. Many studies have proven that this area facilitates, reinforces, and organizes the proper achievement of CRs. According to available information, the full integrity of the hippocampus is needed in order to acquire the conditioning (Weiss et al., 1999; Gruart et al., 2015).

In the present work, classical eyeblink conditioning in rats was achieved using a trace paradigm in which both the CS and the US were electric pulses. Although rats in both paired (conditioned) and unpaired (pseudo-conditioned) groups were presented with CS and US electrical stimuli, only rats assigned to the paired group did establish an association between both stimuli and therefore learned the task (Figures 1C, D, 2A), achieving a CRs ratio over 80% from the fifth session on, slightly faster than in other earlier reports (Weiss et al., 1999). This accelerated acquisition may be related to the type of stimulus chosen, since most studies use a sound as CS, and we have used an electric pulse. The use of sound instead of proprioceptive stimulus seems to delay CR acquisition (Múnera et al., 2001). Moreover, our data showed a gradual decrease in the latencies to start the CRs, along with an increase in the duration and amplitude of the response, in line with others (Weiss et al., 1999; Stanton, 2000; Rogers et al., 2001).

Then, to evaluate the effect of classical eyeblink conditioning on the expression of cell adhesion molecules in the hippocampus, levels of L1, PSA-NCAM, and NCAM in hippocampal crude synaptosomes were determined. A few rodent studies have evaluated the possible regulation on NCAM, its polysialylation and/or L1 by hippocampal-dependent training experiences (Sandi et al., 2003; Maćkowiak et al., 2009; Brandewiede et al., 2014; Yamagishi et al., 2018) but none of them included associative motor learning protocols, despite the great attention devoted by neurobiological studies to this type of learning. Our results indicate that L1, NCAM, and PSA-NCAM expression was only modulated when exposure to electrical stimuli was accompanied by associative motor learning. Although stress have been shown to modulate these molecules (Sandi, 2004; Bisaz et al., 2009; Pesarico et al., 2019), as no differences were observed between context-exposed and pseudo-trained groups, the observed expression changes cannot be attributed to context nor to the stress that may accompany eyelid electric stimulation or motor activity within the arena.

### 4.2 Transient nature of hippocampal learning-induced cell adhesion molecules upregulation

Additionally, to determine the evolution of the expression of these molecules throughout the learning curve, the measurement was carried out after 1st (initial acquisition), 3rd (partial acquisition), and 6th (complete acquisition) conditioning sessions.

We report here that L1 and PSA-NCAM upregulation is transient in the hippocampus. Thus, both CAMs were overexpressed after first and third sessions in the contralateral hippocampus of the conditioned animals compared to both its ipsilateral hippocampus and the hippocampi of the control group. However, after the 6th conditioning session both L1 and PSA-NCAM returned to basal levels. This finding is in accordance with other studies relating learning to changes in cell adhesion molecules (Foley et al., 2003a,b; Knafo et al., 2005a,b) or the number of neurons expressing these molecules (Fox et al., 1995, 2000). It has been suggested that the post-translational attachment of PSA-NCAM may contribute to decrease synaptic adhesion, which allows for the synaptic remodeling observed during learning and memory formation (Benson et al., 2000; Ronn et al., 2000). Furthermore, the deletion of PSA in the hippocampus is deleterious for spatial learning in rats (Becker et al., 1996), hence, it is essential for proper memory formation. Our result is in accordance with other studies that had shown a transitory upregulation of hippocampal PSA after different types of learning (Doyle et al., 1992b; Fox et al., 1995; Murphy et al., 1996; O’Connell et al., 1997; Lopez-Fernandez et al., 2007; Rendeiro et al., 2014). Regarding L1, this CAM has a role in different processes such as neurite growing, axonal fasciculation, cell migration, and CNS repair (Jucker et al., 1996; Schachner, 1997). Likewise, after a fear conditioning training, L1 expression is enhanced in the hippocampus (Merino et al., 2000; Sandi et al., 2001), suggesting a relationship between this molecule and some types of learning (Scholey et al., 1993; Arami et al., 1996; Knafo et al., 2005b) as shown by our results. Interestingly, during initial acquisition, the hippocampus showed alterations of these CAMs in the contralateral side to where the US was presented. This finding agrees with the fact that conditioned responses are preferentially formed on the side of US presentation (Gruart et al., 1995; Jiménez-Díaz et al., 2006) and molecular/cellular/synaptic changes induced by conditioning are found mainly in the contralateral (to the US) brain region studied, including the hippocampus (Gruart et al., 2000, 2015; Jiménez-Díaz et al., 2006).

On the other hand, our data showed a completely different pattern for NCAM expression. During the initial and partial conditioning, there were no alterations of its levels, while after conditioning completion (six sessions), a bilateral enhancement of NCAM was observed in the paired group compared to control animals. Previous studies have related NCAM with the progression from a labile memory to a stable, consolidated memory (Doyle et al., 1992a; Scholey et al., 1993; Sandi et al., 1995; Merino et al., 2000). Our results are in agreement with that and, being that this molecule main role is to stabilize synapses, this upregulation after the acquisition of the conditioned response may be related to the structural changes induced in the hippocampus by this type of associative learning (Geinisman et al., 2000, 2001), including an increase of the post-synaptic density and of the number of synaptic boutons (Sytnyk et al., 2006), although further experiments are required to fully prove this hypothesis.

Moreover, NCAM is composed of three molecular components with relative molecular masses of 120, 140, and 180 kD. NCAM-120 does not appear to be expressed in synapses, whereas NCAM-140 is located on both pre- and post-synaptic membranes and NCAM-180 is restricted mostly to post-synaptic sites (Persohn et al., 1989; Persohn and Schachner, 1990; Schuster et al., 2001). In the present work, all forms of NCAM were detected, and no differences in any particular component were found (data not shown).

## 5 Conclusion

In conclusion, the temporal evolution of the three studied adhesion molecules in the hippocampus along the conditioning eyeblink paradigm (i.e., side-specific increase of PSA-NCAM and L1 expression during early/partial acquisition followed by bilateral increase of NCAM levels when the learned response had been acquired) could account for initial facilitation of structural changes during learning acquisition (by PSA-NCAM and L1) and late stabilization of previously restructured synapses to progress from a labile to a stable motor memory (by NCAM). These results also suggest that CAMs are learning-modulated molecules whose expression needs to be orchestrated in a coordinated manner in the hippocampus for the proper acquisition of hippocampal-dependent associative motor learning.

## Data availability statement

The raw data supporting the conclusions of this article will be made available by the authors, without undue reservation.

## Ethics statement

This animal study was reviewed and approved by Ethical Committee for Use of Laboratory Animals of the National Distance Education University (UNED).

## Author contributions

LJ-D, JD-G, AG, and CS were responsible for the initial conceptualization. LJ-D, AH, KT, KC, and CV performed the experiments. LJ-D, JN-L, and AC analyzed the data, did the writing—review and editing, and were responsible for writing the original draft. CS contributed to the materials. LJ-D, JN-L, JD-G, and AG were responsible for funding acquisition. All authors contributed to the article and approved the submitted version.
