# Supplementary material for: Acquisition-dependent modulation of hippocampal neural cell adhesion molecules by associative motor learning
Source: Front Neuroanat. 2022 Dec 21;16:1082701. doi: 10.3389/fnana.2022.1082701 (PMC9811386; doi:10.3389/fnana.2022.1082701)
Supplement: Supplementary file 1 [file Table_1.pdf]

Supplementary Table 1. Effects of context or unpaired CS-US stimulation on neuronal cell adhesion molecules expression in the hippocampus.

SESSION	SIDE	GROUP	PSA-NCAM	L1	NCAM
1st	ipsi	context	0.217 ± 0.005	0.353 ± 0.015	0.665 ± 0.025
		unpaired	0.206 ± 0.010	0.356 ± 0.004	0.643 ± 0.009
	contra	context	0.215 ± 0.014	0.357 ± 0.017	0.623 ± 0.029
		unpaired	0.213 ± 0.011	0.358 ± 0.005	0.625 ± 0.017
3rd	ipsi	context	0.203 ± 0.009	0.351 ± 0.012	0.676 ± 0.016
		unpaired	0.198 ± 0.008	0.348 ± 0.006	0.656 ± 0.014
	contra	context	0.215 ± 0.006	0.349 ± 0.011	0.640 ± 0.023
		unpaired	0.210 ± 0.006	0.355 ± 0.009	0.628 ± 0.014
6th	ipsi	context	0.195 ± 0.008	0.352 ± 0.004	0.665 ± 0.010
		unpaired	0.198 ± 0.012	0.352 ± 0.005	0.646 ± 0.021
	contra	context	0.201 ± 0.010	0.349 ± 0.009	0.665 ± 0.019
		unpaired	0.196 ± 0.008	0.349 ± 0.008	0.643 ± 0.018

Results are determined by ELISAs in hippocampal synaptosomes and expressed as the optical density obtained by measuring absorbance at 492 nm. Data is expressed as the mean ± SEM for each session (1st: unpaired group n = 6 and context group n = 4; 3rd: unpaired group n = 6 and context group n = 5; 6th: unpaired group n = 6 and context group n = 4). ipsi: ipsilateral hippocampus (to CS-US presentation); contra: contralateral.
